# Fatty acid composition and desaturase gene expression in flax (*Linum usitatissimum* L.)

**DOI:** 10.1007/s13353-014-0222-0

**Published:** 2014-05-29

**Authors:** Dinushika Thambugala, Sylvie Cloutier

**Affiliations:** 1 Department of Plant Science, University of Manitoba, 66 Dafoe Rd, Winnipeg, MB Canada R3T 2N2; 2 Cereal Research Centre, Agriculture and Agri-Food Canada, 195 Dafoe Rd, Winnipeg, MB Canada R3T 2M9; 3 Present Address: Eastern Cereal and Oilseed Research Centre, K.W. Neatby Building, 960 Carling Ave, Ottawa, ON Canada K1A 0C6

**Keywords:** *fad*, Fatty acid composition, Fatty acid desaturase, Flax, Gene expression, Promoter analysis, *sad*

## Abstract

Little is known about the relationship between expression levels of fatty acid desaturase genes during seed development and fatty acid (FA) composition in flax. In the present study, we looked at promoter structural variations of six FA desaturase genes and their relative expression throughout seed development. Computational analysis of the nucleotide sequences of the *sad1*, *sad2*, *fad2a*, *fad2b*, *fad3a* and *fad3b* promoters showed several basic transcriptional elements including CAAT and TATA boxes, and several putative target-binding sites for transcription factors, which have been reported to be involved in the regulation of lipid metabolism. Using semi-quantitative reverse transcriptase PCR, the expression patterns throughout seed development of the six FA desaturase genes were measured in six flax genotypes that differed for FA composition but that carried the same desaturase isoforms. FA composition data were determined by phenotyping the field grown genotypes over four years in two environments. All six genes displayed a bell-shaped pattern of expression peaking at 20 or 24 days after anthesis. *Sad2* was the most highly expressed. The expression of all six desaturase genes did not differ significantly between genotypes (P = 0.1400), hence there were no correlations between FA desaturase gene expression and variations in FA composition in relatively low, intermediate and high linolenic acid genotypes expressing identical isoforms for all six desaturases. These results provide further clues towards understanding the genetic factors responsible for FA composition in flax.

**Electronic supplementary material:**

The online version of this article (doi:10.1007/s13353-014-0222-0) contains supplementary material, which is available to authorized users.

## Introduction

Flax (*Linum usitatissimum* L.) is the leading source of plant-based omega-3 fatty acids (FAs) praised for their health benefits in humans and animals. Oilseed flax, also known as linseed, generally contains 40-50 % oil and its quality is largely determined by its FA composition (Green 1986; Cloutier et al. 2010). Linseed oil is primarily composed of palmitic (PAL, C16:0; ∼6 %), stearic (STE, C18:0 ∼ 4.4 %), oleic (OLE, C18:1 ∼ 24.2 %), linoleic (LIO, C18:2 ∼ 15.3 %) and linolenic (LIN, C18:3 ∼ 50.1 %) acids (Muir and Westcott 2003). The high levels of alpha-linolenic acid (ALA or LIN) and moderate levels of LIO in linseed oil not only contribute to a healthy diet but are considered essential FAs because humans lack the ∆^12^ and ∆^15^ desaturase enzymes that convert OLE to LIO and LIO to LIN, respectively (Damude and Kinney 2008). Humans can use these FAs as substrates for further elongation and desaturation leading to the formation of very long chain polyunsaturated FAs (VLCPUFAs) like ecosapentaenoic acid (EPA, C22:5), docosahexaenoic acid (DHA, C22:6) and arachidonic acid (AA, C20:4) (Warude et al. 2006). These VLCPUFAs also have health benefits and studies have established their important role in reducing total and low-density lipoprotein (LDL) cholesterol levels in humans and preventing chronic diseases including cardiovascular diseases, hormonal cancers and arthritis (Oomah 2001; Wiesenfeld et al. 2003; Ander et al. 2004; Dyer et al. 2008). However, the high LIN content of flaxseed oil makes it more susceptible to oxidation and rancidity (Zuk et al. 2012), thus limiting its use as an edible oil, but simultaneously providing it with unique drying properties that makes it valuable in various industrial applications (Green 1986).

Genetic control of FA biosynthesis in flax has been studied and many of the genes encoding the enzymes that perform FA synthesis have been identified and characterized (Green 1986; Fofana et al. 2004, 2006; Sorensen et al. 2005; Vrinten et al. 2005; Krasowska et al. 2007; Khadake et al. 2009; Banik et al. 2011; Thambugala et al. 2013). FA desaturation and elongation are important biochemical processes that drive the multi-step FA biosynthetic pathway in a sequential manner, leading to synthesis of polyunsaturated FAs (Warude et al. 2006; Khadake et al. 2011). Fatty acid desaturases (FADs) are the key enzymes that introduce double bonds into FA acyl chains in a stepwise manner starting from STE (Los and Murata 1998; Shanklin and Cahoon 1998; Smooker et al. 2011). The desaturation of STE is sequentially catalyzed by desaturases namely, stearoyl-ACP desaturase (SAD) (Singh et al. 1994; Jain et al. 1999), fatty acid desaturase 2 (FAD2) (Krasowska et al. 2007; Khadake et al. 2009) and fatty acid desaturase 3 (FAD3) (Vrinten et al. 2005; Banik et al. 2011). In flax, these three enzymes are encoded by duplicated genes (Fofana et al. 2010). The two FAD3 enzymes, FAD3A and FAD3B, have been shown to be the major enzymes controlling LIN content in linseed (Vrinten et al. 2005).

Although the genetic variability of desaturase genes and their impact on FA composition in flax have been studied (Thambugala et al. 2013), only two studies on the regulation and expression of *sad* and *fad* genes during seed development have been reported (Fofana et al. 2006; Banik et al. 2011). Fofana et al. (2006) reported that the expression of *sad* and *fad2* genes in flax was modulated during seed development whereas Banik et al. (2011) found that the expression patterns of *fad3a* and *fad3b* were highly correlated with LIN accumulation during seed development.

In our previous study, we characterized the genetic variability for *sad1, sad2, fad2a, fad2b, fad3a* and *fad3b* genes in flax by sequencing the six genes from 120 flax accessions (Thambugala et al. 2013). Between five and 21 alleles corresponding to two to seven isoforms were identified for the six desaturases. Thirty-four accessions had an identical isoform composition for all six desaturase genes but their FA composition varied significantly. We hypothesized that FA composition differences in these lines could result from differential expression of the desaturase genes during seed development. Based on this hypothesis, this study had three goals. First, to quantify the expression levels of the desaturase genes at different stages of seed development by semi-quantitative reverse transcriptase (RT)-PCR in relatively low, intermediate and high LIN genotypes expressing identical isoforms for all six desaturases. Second, to study the structural differences in the promoter region of the six desaturase genes. Third, to correlate these structural and expression data with FA composition as determined by phenotyping the field grown genotypes during four years at two locations with the overall objective of gaining a greater understanding of the genetic factors controlling the FA composition in flax.

## Materials and methods

### Plant material

FA composition of 34 flax accessions carrying identical isoforms for the desaturase genes *sad1, sad2, fad2a, fad2b, fad3a* and *fad3b* were analysed as previously described (Thambugala et al. 2013) and six linseed genotypes showing significantly different (P < 0.05) FA profiles were selected for this study (ESM 1). These six genotypes, including two high (UGG5-5, M5791), two intermediate (FP2270, CN30861) and two relatively low LIN (CN97334, CN97407), were grown in a growth chamber under the following conditions: 22 °C with a 16-h photoperiod at a photon density of approximately 145 μE · m^– 2^ · s^– 2^ (Fofana *et al*. 2004). Flowers were tagged at anthesis and developing bolls harvested at 8, 12, 16, 20, 24, 28 and 32 days after anthesis (DAA) were immediately frozen in liquid nitrogen where they were stored until RNA extraction.

### RNA extraction

Total RNA was extracted from 8-32 DAA developing bolls of each genotype using the RNA extraction procedure described in Banik et al. (2011). For each extraction, 0.2 g of bolls were used. Final total RNA pellets were resuspended in 50 μl RNase-free water and stored at –80 °C. The RNA was quantified by nano-spectrophotometer (Implen GmbH, Munich, Germany).

### First strand cDNA synthesis

To remove any potential remnant DNA, total RNA from each developmental stage and genotype was treated with TURBO DNase according to the manufacturer’s instructions (Ambion, Austin, Texas, USA). The DNase treated RNA was used as a template to synthesize first strand cDNA using oligo(dT) primer and Superscript^TM^ II reverse transcriptase followed by RNaseH treatment as per the manufacturer’s recommendations (Invitrogen, Carlsbad, CA, USA). An amount of 800 ng total RNA was used to synthesize the cDNA in three independent 20 μl reactions for each developmental stage and each genotype. Pooled cDNA samples of each developmental stage and genotype were stored at –20 °C.

### cDNA quantification

A fluorometric method was used to precisely quantify the first strand cDNA. In this method, the RNA was digested and the single-stranded cDNA was quantified by fluorescence using RiboGreen (Invitrogen) as described by Libus and Storchová (2006). cDNA quantification was performed in duplicate for each development stage of each genotype.

### RT-PCR of *sad* and *fad* genes

Semi-quantitative RT-PCR was performed using the quantified cDNA samples from 8-32 DAA using 28 cycles as previously recommended (Kumar et al. 2013). Gene-specific PCR primers (Table 1) for *sad1, sad2, fad2a, fad2b, fad3a* and *fad3b* were designed using Primer Express (Applied Biosystems) and Primer 3 software (Rozen and Skaletsky 2000). Optimized amplification reactions (25 μL) contained 4 ng cDNA, 1X PCR buffer, 1.5 mM MgCl_2_, 0.8 mM dNTPs, 0.4 μM each primer and 1.5 U Taq DNA polymerase. The PCR reactions were first denatured at 94 °C for 5 min followed by 28 cycles consisting of 94 °C for 30 s; 62 °C for 30 s and 72 °C for 60 s, prior to a final extension at 72 °C for 10 min. RT-PCR products were resolved on 2.5 % agarose gels stained with ethidium bromide. The flax adenine phosphoribosyl-transferase 1 (*apt1*) gene was used as a reference control in all RT-PCR experiments (Banik et al. 2011). Three independent RT-PCR replications were performed for each gene, each developmental stage and genotype, including *apt1*. The expression of the target genes relative to the reference gene *apt1* was evaluated by densitometric analysis of the signal strength of the semi-quantitative RT-PCRs with the AlphaImagerHP software (version 3.4, proteinsimple, Santa Clara, CA, USA). Desaturase gene to *apt1* ratios were calculated by dividing the background corrected signal of the PCR amplicon of the desaturase gene to that of *apt1*.

**Table 1:** Sequences and melting temperature of primers used for semi-quantitative RT-PCR and promoter analysis (amplification and sequencing)

Gene	Primer name	Sequence (5′ to 3′)	Tm (°C)
*apt1*	APT1-319 F	TAGAGCTGACCAGGACAAACA	62
	APT1-409R	GTTTATGAATGCGCTTGTCTCA	62
*sad1*	SAD1-F770	TCGCAGCAGACGAGAAACG	60
	SAD1-R840	AGGGTCGATCTCGAAGAGCTT	58
*sad2*	SAD2-F202	AAGCTGGAGATCTTTAAGTCCCTTGA	59
	SAD2-R307	GTTCGGGCAGGAAATCTTGT	58
*fad2a*	FAD2A-F873	CGTGGATCGAGACTACGGGTTA	60
	FAD2A-R942	ATGGTGCGCGACATGTGT	58
*fad2b*	FAD2B-F496	TGGCACTCAAAGTACCTCAACAA	58
	FAD2B-R571	AAGGCCAGCCGAGAGTGA	58
*fad3a*	FAD3A-F40	GACTTCAAAACTGTGGCTCT	55
	FAD3A-R132	GATAGCCACACCATTGGTGC	62
*fad3b*	FAD3B-F662	GCAGCGGTCTTGATTTCAACA	48
	FAD3B-R759	ATTTTGAGGACCGGAGCGAA	50
Promoter analysis			
*sad1*	SAD1-F53350	AATGCCTCCAAAGTGCTCTC	59
	SAD1-R54516	GCTTACTTGGTGGAGGTGGA	60
	SAD1-F53628	TTTGGTGACTCGAAAGTTCT	55
	SAD1-R53974	CATATGACATTGCAAGACGA	56
*sad2*	SAD2-F10585	CGTCCCAATTGATGACAATG	60
	SAD2-R11671	TGGAATTGAAAGTGGAAGCA	59
	SAD2-F10975	CCAAAGTGCTCTCTACTTGC	55
	SAD2-R11296	GCGTTTCATCAGTTCTATCG	56
*fad2a*	FAD2A-F22	CGGCGATTTTGAAGTGCAT	62
	FAD2A-R868	CTCACCGAGCGTGAATGGT	62
	FAD2A-F252	GCCCTCCTTCATATTCTTCT	55
	FAD2A-R566	TCCTTTCCAGTTTTCAGTTG	55
*fad2b*	FAD2B-F1036	AAGGGTGATGGTCTTGATGC	60
	FAD2B-R2094	AGGGACGGCTTTCTTGATCT	60
	FAD2B-F1239	TACCCTAAAGTGATCAATGG	53
	FAD2B-R1569	AGCAAGTAGTGCTATCCTGA	53
*fad3a*	FAD3A-F949	TCGATTGCAAAGCAAGAGAG	59
	FAD3A-R2018	AACGGCGAAGCTGAGGAT	61
	FAD3A-F1228	TTAGTCGATTTCACCCTAGC	55
	FAD3A-R1503	AACGGTTGTTGTTACTTGCT	55
*fad3b*	FAD3B-F15266	CCCAACCCATTACATGACG	60
	FAD3B-R16252	GCTGAGGATGACAAGGAGGT	59
	FAD3B-F15435	AACATTGCAATTCAGAGTCC	55
	FAD3B-R15777	TCTGCTCTTTATTGGGTTTC	55

### Promoter analysis of *sad* and *fad* genes

Promoter sequences corresponding to bases -800 to +200 relative to the transcription start site (TSS) of *sad* and *fad* genes were obtained from the six flax genotypes by sequencing the amplicons with Big-Dye V3.1 Terminator chemistry and resolving them on an ABI 3130xl Genetic Analyser (Applied BioSystems, Foster City, CA, USA). All sequences were processed using PHRED (Ewing et al. 1998) and assembled with CAP3 (Huang and Madan 1999) as implemented in the internal data pipeline called SOOMOS v0.6 (T. Banks, personal communication). Sequence alignments were performed with Clustal W (Thompson et al. 1994). TSSs were identified using the bioinformatics pipeline for TSS signals (http://fruitfly.org/seq_tools/promoter.html) (Reese 2001). Promoter analysis was performed with PLAnt Cis-acting regulatory DNA Elements (PLACE) (Higo et al. 1999) and PLANT Promoter Analysis Navigator (PlantPAN) (http://plantpan.mbc.nctu.edu.tw/seq_analysis.php) (Chang et al. 2008).

### Fatty acid composition

Field plots of the 34 flax accessions were grown in a type 2 modified augmented design (MAD) (Lin and Poushinsky 1985) at the Kernen farm near Saskatoon (SK, Canada) and at the Morden Research Station (MB, Canada) in 2009, 2010, 2011 and 2012 (ESM 1). FA composition and oil content (OIL) were obtained from all eight field experiments as previously described (Thambugala et al. 2013).

### Statistical analysis

The phenotypic data for FA composition and OIL (ESM 1) were adjusted for soil heterogeneity based on the MAD statistical analysis method using the recently described pipeline (You et al. 2013). Variance components were calculated using adjusted phenotypic data to determine the effect of year, location, genotype and their interactions on FA composition and OIL using the PROC GLM procedure (SAS Institute, Cary, NC, USA). To assess the differences in FA composition among the six genotypes, one-way analysis of variance (ANOVA) was used followed by the Duncan’s multiple range comparison test at 0.05 probability level. A similar analysis was performed to determine statistical significance between the ratios of FA desaturase gene to *apt1* of the six desaturase genes, across all six genotypes and for all seven stages of seed development. All statistical analyses were carried out using SAS v9.2 (SAS Institute, Cary, NC, USA).

## Results

### Fatty acid composition

**Fig. 1:**
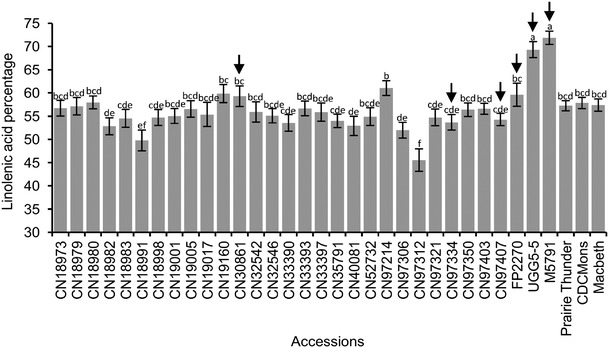
Linolenic acid content of 34 flax accessions carrying identical isoforms for the fatty acid desaturases SAD1, SAD2, FAD2A, FAD2B, FAD3A and FAD3B. The sample means were averaged from two locations (MB and SK) over four years (2009, 2010, 2011 and 2012). Arrows indicate the accessions selected for the fatty acid desaturase gene expression study. Error bars represent the standard error of the mean. Letters above the bars indicate statistical significance of the Duncan’s multiple range tests

**Fig. 2:**
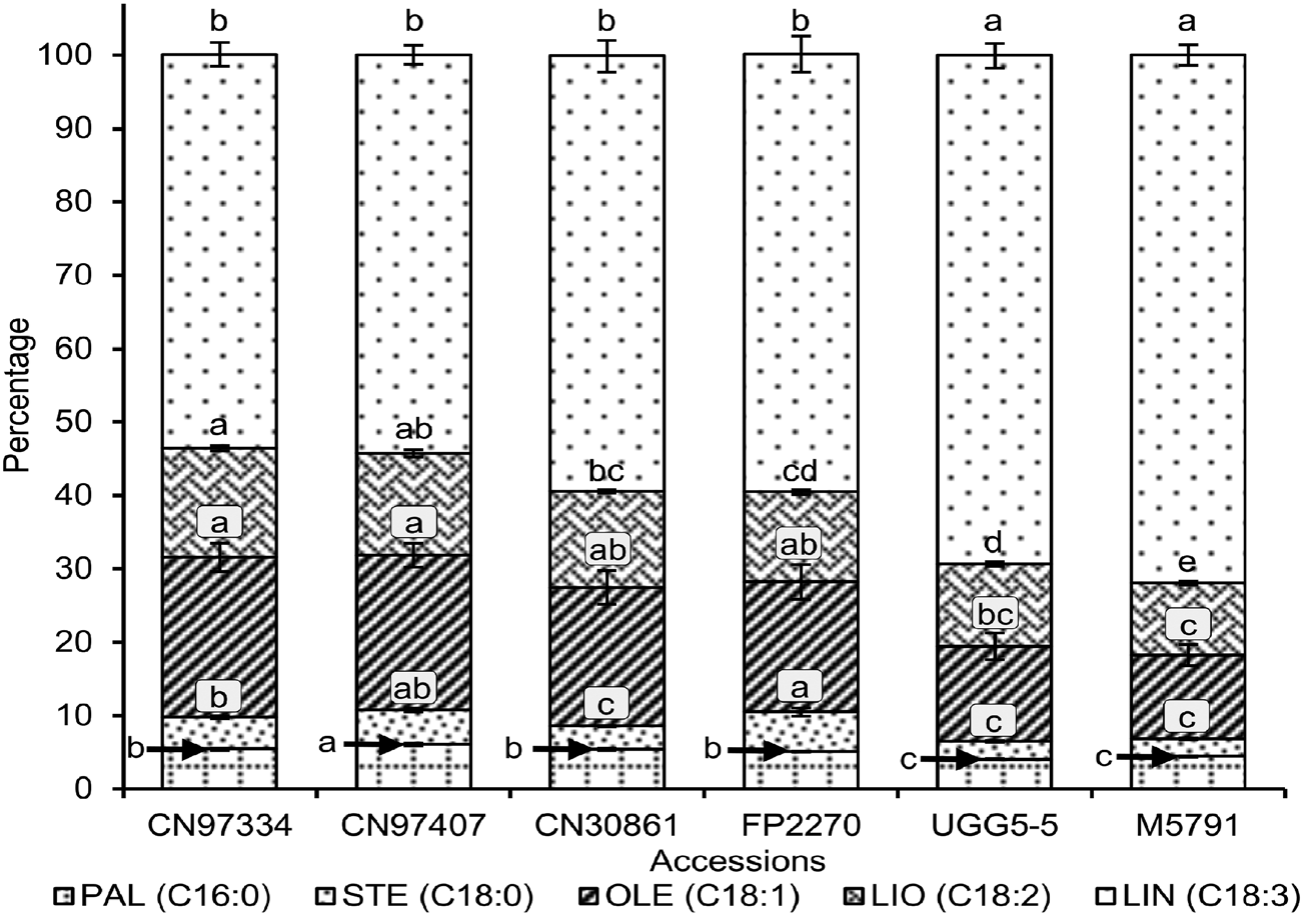
Fatty acid composition of CN97334, CN97407, CN30861, FP2270, UGG5-5 and M5791. Percentages of the five main fatty acids, namely palmitic (PAL, C16:0), stearic (STE, C18:0), oleic (OLE, C18:1), linoleic (LIO, C18:2) and linolenic (LIN, C18:3) acids for each genotype are illustrated. Error bars represent standard error of the mean. Letters beside or above the bars indicate statistical significance of the Duncan’s multiple range tests

The FA composition of the 34 flax accessions with identical isoform composition for all six desaturase genes displayed significant variations (P < 0.0001) in LIN content ranging from 46 to 72 % (Fig. 1). Six of those accessions were selected on the basis of their significantly different (P < 0.05) FA compositions. All five FAs showed significant differences across the six genotypes (Fig. 2). UGG5-5 and M5791 had high LIN but low LIO content. In contrast, CN97334 and CN97407 had lower LIN but higher LIO than UGG5-5 and M5791. Significant FA compositional variations were also found for PAL, STE and OLE (Fig. 2).

### *Sad* and *fad* gene expression during seed development

**Fig. 3:**
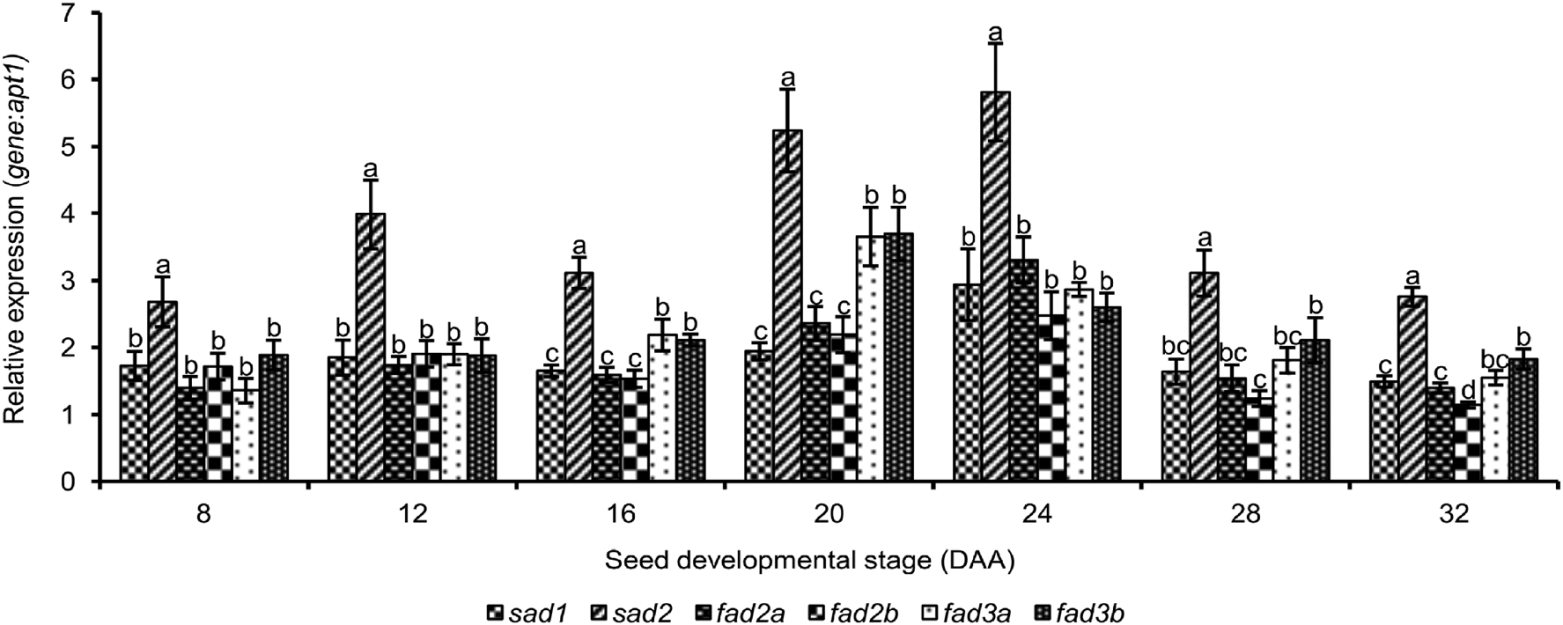
Relative expression of the fatty acid desaturase genes *sad1, sad2, fad2a, fad2b, fad3a* and *fad3b* in flax during seed development. Error bars represent standard error of the mean and are based on three independent RT-PCR replicates. Letters above the bars indicate statistical significance of the Duncan’s multiple range tests

A semi-quantitative RT-PCR method was used to study expression of the six FA desaturases during the seed developmental stages of flax from 8-32 DAA in six flax genotypes (ESM 2). With the exception of *sad1*, expression was significantly modulated (P < 0.0001) for the other desaturases during seed development (ESM 3). All six genes followed a similar pattern where gene expression tended to increase from eight to 20 DAA, peaked at 20 or 24 DAA and decreased during the later stages of seed maturation (Fig. 3). *Sad2* was the most highly expressed gene (ESM 2) throughout all stages of seed development, peaking at 24DAA (Fig. 3). *Sad1* expression was lower than *sad2* and generally remained more constant throughout all developmental stages (Fig. 3).

*Fad2* and *fad3* displayed a similar pattern where gene expression increased from eight to 24DAA and 20DAA respectively and decreased towards maturity. Over all seed developmental stages, *sad* and *fad* expression were not significant between genotypes (P = 0.1400) (ESM 2, ESM 3). However, at 32 DAA, significant differential expression between genotypes was observed for *fad2a*, *fad3a* and *fad3b* (ESM 4). *Fad2a* and *fad3a* were more highly expressed in the high-LIN line M5791 whereas *fad3b* was expressed at a lower level in FP2270 than in the other five genotypes (Table 2).

**Table 2:** Expression of desaturase genes *sad1, sad2, fad2a, fad2b, fad3a* and *fad3b* during seed development of six flax genotypes

Seed developmental stage (DAA)	Genotype	Relative expression (*gene*:*apt1*)					
		*sad1*	*sad2*	*fad2a*	*fad2b*	*fad3a*	*fad3b*
8	CN97334	1.0	1.9	0.8	1.4	0.9	1.3
	CN97407	2.1	3.8	1.5	2.0	1.4	2.0
	CN30861	1.6	3.3	1.4	2.1	1.8	2.3
	FP2270	2.0	2.3	1.3	1.9	0.8	1.9
	UGG5-5	1.7	2.2	1.2	1.3	1.2	1.4
	M5791	1.9	2.6	2.6	1.7	2.1	2.4
12	CN97334	1.4	3.1	1.4	1.6	1.4	1.6
	CN97407	2.0	4.3	2.0	1.9	2.2	2.3
	CN30861	1.7	4.2	1.8	1.9	2.2	2.1
	FP2270	1.7	3.7	1.5	1.7	1.7	1.9
	UGG5-5	2.2	4.6	2.1	2.4	2.1	1.9
	M5791	2.1	4.1	1.7	1.9	1.8	1.6
16	CN97334	1.6	2.3	1.7	1.5	1.5	2.0
	CN97407	1.4	2.6	1.5	1.4	2.2	2.0
	CN30861	2.1	4.1	1.9	1.8	3.2	2.5
	FP2270	1.6	3.3	1.7	1.6	2.4	2.0
	UGG5-5	1.5	3.2	1.4	1.3	2.1	2.1
	M5791	1.8	3.2	1.3	1.6	1.7	2.1
20	CN97334	1.9	5.1	2.2	2.4	3.0	3.2
	CN97407	1.9	6.2	2.0	2.2	3.7	4.1
	CN30861	2.2	6.0	3.0	2.7	4.5	4.4
	FP2270	1.8	5.2	2.1	2.1	3.7	3.7
	UGG5-5	1.7	4.6	2.4	1.8	3.5	3.5
	M5791	2.2	4.3	2.4	2.0	3.6	3.3
24	CN97334	3.1	5.9	3.8	2.8	2.8	2.4
	CN97407	2.7	5.5	3.0	2.0	2.6	2.3
	CN30861	3.0	6.5	3.0	2.6	3.0	2.3
	FP2270	2.8	6.1	3.2	2.4	2.8	2.7
	UGG5-5	2.7	5.0	3.4	2.3	3.1	3.1
	M5791	3.3	6.0	3.4	2.8	2.9	2.8
28	CN97334	1.6	3.8	1.4	1.3	1.6	3.0
	CN97407	1.4	3.3	1.5	1.1	1.5	2.1
	CN30861	1.7	4.0	1.6	1.5	2.2	2.4
	FP2270	1.9	2.9	1.4	1.4	2.0	2.0
	UGG5-5	1.7	3.2	1.7	1.3	1.9	1.8
	M5791	1.5	1.6	1.7	0.9	1.7	1.3
32	CN97334	1.4	2.9	1.3^b^	1.2	1.5^b^	2.0^a^
	CN97407	1.3	2.8	1.3^b^	1.1	1.6^ab^	2.2^a^
	CN30861	1.6	2.5	1.3^b^	1.2	1.6^ab^	2.3^a^
	FP2270	1.6	3.1	1.3^b^	1.2	0.8^c^	0.7^b^
	UGG5-5	1.4	2.6	1.3^b^	0.9	1.8^ab^	1.6^a^
	M5791	1.7	2.7	1.9^a^	1.3	2.1^a^	2.0^a^

^a,b,c^ Statistical significance (*p* < 0.05) of Duncan’s multiple range tests among genotypes within each gene and developmental stage

### Promoter analysis

Promoter sequence analysis of *sad* and *fad* genes corresponding to bases from -800 to +200 relative to the TSS revealed single point mutations in the promoter region of *sad1*, *sad2* and *fad3b* of CN30861 (ESM 5). Computational analysis of promoter regions of the six desaturase genes indicated the presence of several basic transcriptional elements such as CAAT and TATA boxes. In addition to these basic elements, many seed or endosperm specific and ABA-responsive cis-elements (ABRE) including several Dof core (AAAG motif), DPBP core (ACACNNG motif), E-box (CANNTG motif), Myb-core (CNGTTR motif) and ACGT-box (AACGTT/ABRE motif) were identified. The sequence analysis also revealed the presence of motifs similar to pollen-specific cis-acting elements; POLLEN1LELAT52 (AGAAA) is one of two co-dependent regulatory elements responsible for the pollen-specific activation of genes (ESM 5).

### Phenotypic data

All FA traits showed significant genotype (G), location (L) and year (Y) effects (P < 0.001; ESM 6). Genotype by environment interactions (GEs: G*L, G*Y, L*Y and G*L*Y) were also significant for all FA composition traits and oil content (ESM 6).

## Discussion

FADs display significant diversity in their sequences and expression (Los and Murata 1998; Warude et al. 2006) and hence are considered biotechnological targets for manipulation of FA composition of oilseed crops (Khadake et al. 2009). Although the genetic variability for FADs and their impact on FA composition in flax has been recently reported (Thambugala et al. 2013), little is known about how the *fad* expression levels during seed development affect FA composition. In the present study, expression patterns of *sad1, sad2, fad2a, fad2b, fad3a* and *fad3b* of six flax genotypes at various seed developmental stages were studied using semi-quantitative RT-PCR analysis.

Semi-quantitative RT-PCR is a highly sensitive and specific method to analyse the expression of genes (Choquer et al. 2003). The reliability of this method depends on a number of factors including RNA quality, primer specificity, technique precision and use of a stable house-keeping gene (Wong and Medrano 2005; Banik et al. 2011). Although semi-quantitative RT-PCR is mostly used as a qualitative method of analysing gene expression, the determination of both *fad* and *apt1* reference gene products by densitometric analysis enabled quantification and permitted comparisons across developmental stages, genes and genotypes (Choquer et al. 2003; Libus and Storchová 2006). The *Arabidopsis apt1* gene has been identified as one of the most stable internal controls (Gutierrez et al. 2008) and the flax *apt1* ortholog used in this study confirmed its consistent expression across all seed developmental stages and genotypes (Livak and Schmittgen 2001; Pfaffl 2005; Banik et al. 2011). Furthermore, the approach of quantifying cDNA precisely with the RiboGreen method reduces the variability associated with variations in starting material, hence adding precision to the evaluation method (Libus and Storchová 2006).

To establish correlations between gene expression and variation in FA composition, we examined expression patterns of *sad* and *fad* genes using six flax genotypes varying in LIN content. To our knowledge, this is the first report in flax comparing expression levels of all six desaturase genes and FA composition during seed development from genotypes that differed significantly in LIN content. Oil accumulation is a highly controlled developmental process. Genetic studies indicated that genes of the FA biosynthetic pathways, including triacylglycerol (TAG) synthesis, are regulated at the level of transcription (Baud and Graham 2006; O’Hara et al. 2002; Ohlrogge and Jaworski 1997). Gene expression programs related to FA synthesis are activated during the maturation phase and most genes encoding FA synthesis enzymes display a bell-shaped pattern of expression during seed development (Baud and Lepiniec 2009). Similarly, the six FA desaturases studied herein all displayed the bell-shaped pattern of expression with a peak at or after 20 DAA. Although the flax genome contains two paralogous *sad* loci, *sad1* and *sad2*, they are differentially expressed (Jain et al. 1999) with *sad1* having lower and more constant expression throughout seed development. The highly conserved nature of *sad2* and its higher expression are in agreement with its essential ∆9-desaturase role in the lipid biosynthetic pathway in flax (Allaby et al. 2005; Thambugala et al. 2013).

Transcriptional control of *fad* gene expression in flax has been demonstrated (Fofana et al. 2006; Banik et al. 2011). The two paralogous *fad2a* and *fad2b* genes have been cloned and characterized from flax (Krasowska et al. 2007; Khadake et al. 2009). Both genes displayed relatively low but steady expression patterns throughout seed development. Fofana et al. (2006) demonstrated the seed-specific expression of *fad2a* while constitutive expression of *fad2b* has also been reported (Cao et al. 2013; Schlueter et al. 2007). *Fad3a* and *fad3b* had similar expression patterns as the other four desaturase genes except that their expression peaked at 20 DAA instead of 24 as previously shown for other flax accessions by Banik et al. (2011) who also demonstrated that the *fad3* expression correlated with LIN accumulation during seed development.

*Fad2* genes are thought to be the rate-limiting genes of the FA biosynthesis pathway in flax and are highly influenced by the environment (Fofana et al. 2006). The significant GE interaction observed for FA traits also suggests the complex interactions of gene and environmental cues on FA composition. Temperature during the growing season affects FA composition of flax and other oil crops (Casa et al. 1999; Fofana et al. 2006; Baud and Lepiniec 2010). The higher thermal stability of safflower’s FAD2 compared to that of sunflowers was proposed to explain the more stable FA composition of safflower irrespective of the temperature during seed development (Esteban et al. 2004). Effects of varying temperature on *sad* gene expression alters the FA composition of soybean seeds (Byfield and Upchurch 2007).

Identification of molecular components that regulate expression patterns of FA biosynthesis genes is important for understanding the variation in FA composition in flax. Activators and repressors fine-tune the expression level of FA biosynthetic pathway genes (Bene et al. 2001; Baud and Lepiniec 2010; Saed Taha et al. 2012). An AW-box sequence [CnTnG](n)_7_[CG] identified in the promoter regions of several FA biosynthetic genes was proposed to be recognized by WRI1, a transcription factor responsible for activating these genes (Maeo et al. 2009; Baud et al. 2007). Although promoters of the six desaturase genes share a functionally similar promoter core, their expression can be modulated by differences in upstream regulatory elements. Kim et al. (2006) reported that the strong seed specific expression of the sesame *fad2* gene is controlled by negative cis-regulatory elements of the promoter and enhancers located in the 5′-UTR (untranslated region). Temporal control of *fad2* via ABA-response elements was also reported (Kim et al. 2006). However, the functions of these putative regulatory elements for the expression of FAD genes in flax are yet to be determined.

Millar and Kunst (1999) reported that the natural genetic variation of the FA composition of seed oils in different ecotypes of *Arabidopsis thaliana* are probably due to altered expression levels or activities of FA biosynthetic enzymes. Here, we looked at the expression levels of six desaturase genes in six genotypes that differed for FA composition but that encoded the same desaturase isoforms and we were unable to establish correlations between the expression of any of the desaturase genes with the FA composition of the six flax genotypes. Two main hypotheses provide potential explanations for our results. First, genetic factors other than the six desaturases studied here may play an important role in determining the FA composition of flax. Although desaturases and thioesterases are the major enzymes responsible for FA composition in oilseed crops (Ohlrogge and Jaworski 1997; Baud and Lepiniec 2010), minor genes may play an important role in determining the FA composition variation. Complete genome sequences of many plant species have indeed allowed the identification of a number of genes involved in plant oil biosynthesis (Ying et al. 2012). However, the factors leading to variations in FA composition remain to be fully understood (Hobbs et al. 2004; Keurentjes et al. 2006). Genes of minor effect have generally been considered responsible for variation observed in LIN content in several oil crops including flax (Das and Rai 1974; Doucet and Filipescu 1981; Green 1986; Cloutier et al. 2010), rapeseed (Kondra and Thomas 1975; Pleines and Friedt 1989) and soybean (White et al. 1961). Green (1986) reported that the small differences in LIN content between ‘Glenelg’ and the majority of current flax varieties are most probably due to the cumulative effects of several minor genes that modify the expression of *fad3a* and *fad3b* (Vrinten et al. 2005). QTL and association mapping may help in identifying minor genes controlling FA composition (Soto-Cerda et al. 2013). Of these putative minor genes, Lei et al. (2012) suggested that acyl carrier protein (ACP), 3-ketoacyl-ACP-synthase (KAS) and acyl-ACP thioesterase (FATA) play a role in FA composition and may also be rate limiting. ACP, KAS and FATA gene expression correlated significantly with monounsaturated FA and PUFA synthesis (Lei et al. 2012). Similarly, natural variation in long chain FA content in *Arabidopsis thaliana* was found to be controlled by a new isoform of β-ketoacyl-CoA synthase 18 (KCS18; Jasinski et al. 2012).

The second hypothesis is that differential expression of desaturases can indeed explain FA compositional differences in lines with identical isoforms grown in the field but that we could not demonstrate it because we measured the expression under controlled conditions. Environment and genotype by environment significantly affect FA composition (Deng and Scarth 1998; Hernández et al. 2011). The effect of the environment on *fad2* expression in flax has been documented (Fofana et al. 2006). It is therefore conceivable that correlations may exist between desaturase expression levels and FA composition if the former was measured in the same field environment as the latter. This second hypothesis is not mutually exclusive to the minor genes hypothesis because minor genes could be responsible for translating the environmental cues that affect expression of desaturases. Further studies of desaturase gene expression in field environments combined with phenotyping of the genotypes in the same environments are needed to elucidate and partition the role of the genetic and environmental factors in FA composition in flax.

Seed FA composition has become a major target for modification by plant breeding and genetic engineering in many oil crops for food and non-food purposes (Murphy 1996; Damude and Kinney 2008; Cahoon et al. 2010). A greater understanding of the genetic control of the FA composition of linseed oil is essential to develop flax cultivars producing oils for specific end-uses. The current study provides some thought-provoking hints to understand the genetic components such as transcription factors and genes other than the FA desaturases, controlling FA composition in flax, but further investigations are required to fill the knowledge gap.

## Electronic supplementary material

Below is the link to the electronic supplementary material.

ESM 1 Description of flax accessions used for *sad* and *fad* gene expression study with the predicted *sad* and *fad* alleles and isoforms according to the nomenclature previously described (Thambugala et al. 2013). Phenotypic data for fatty acid composition and oil content were averaged from two locations (MB and SK) over four years (2009, 2010, 2011 and 2012). (PDF 25 kb)

ESM 2 Reverse transcriptase PCR of six fatty acid desaturase genes and the reference *apt1* gene from six flax genotypes comparing the level of expression at 20 days after anthesis showing the consistent amplification of the control *apt1* gene and the relative differential expression of the six desaturase genes. (PDF 22 kb)

ESM 3 Analysis of variance for expression (*gene:apt1*) of six desaturase genes. Mean square values and statistical significance for *sad1, sad2, fad2a, fad2b, fad3a* and *fad3b* during seed development of six flax genotypes are shown. (PDF 92 kb)

ESM 4 *P*-value of *sad1, sad2, fad2a, fad2b, fad3a* and *fad3b* for genotypes during seed development. (PDF 89 kb)

ESM 5 Nucleotide sequences of the promoter region of *sad1*, *sad2*, *fad2a*, *fad2b*, *fad3a* and *fad3b* genes. Sequence numbering is relative to the ATG codon (+1). 5′-UTR is highlighted and putative transcription initiation site (TSS) is indicated. The TATA and CAAT boxes are boxed. Putative *cis*-acting regulatory elements are underlined and designated with the names of each motif (Higo et al. 1999; Chang et al. 2008). Red colour letters indicate the single point mutations identified in the promoter regions of the corresponding genotype. (PDF 198 kb)

ESM 6 Analysis of variance for fatty acid composition for the data collected from six environments. Mean square values and statistical significance for palmitic acid (PAL), stearic acid (STE), oleic acid (OLE), linoleic acid (LIO) and linolenic acid (LIN) are shown. (PDF 10 kb)
