# Supplementary material for: Fatty acid composition and desaturase gene expression in flax (Linum usitatissimum L.)
Source: J Appl Genet. 2014 May 29;55(4):423–32. doi: 10.1007/s13353-014-0222-0 (PMC4185102; doi:10.1007/s13353-014-0222-0)
Supplement: Supplementary file 1 — Description of flax accessions used for sad and fad gene expression study with the predicted sad and fad alleles and isoforms according to the nomenclature previously described (Thambugala et al. 2013). Phenotypic data for fatty acid composition and oil content were averaged from two locations (MB and SK) over four years (2009, 2010, 2011 and 2012). (PDF 25 kb) [file 13353_2014_222_MOESM1_ESM.pdf]

ESM_1. Description of flax accessions used for *sad* and *fad* gene expression study with the predicted *sad* and *fad* alleles and isoforms according to the nomenclature previously described (Thambugala et al. 2013). Phenotypic data for fatty acid composition and oil content were averaged from two locations (MB and SK) over four years (2009, 2010, 2011 and 2012).

Accession description	Accession number	Allele and Isoform												Palmitic acid (PAL)		Stearic acid (STE)		Oleic acid (OLE)		Linoleic acid (LIO)		Linolenic acid (LIN)		Oil (OIL)	
		SAD1		SAD2		FAD2A		FAD2B		FAD3A		FAD3B		%[1]	SE[2]	%	SE	%	SE	%	SE	%	SE	%	SE
Mocoreta	CN97334	2	A	3	A	1	A	1	A	2	A	1	A	5.40	0.17	4.39	0.21	21.76	1.95	14.86	0.40	53.67	1.68	44.18	0.91
Rio (Long 79)	CN97407	2	A	1	A	1	A	1	A	1	A	1	A	6.03	0.22	4.76	0.29	20.99	1.61	13.96	0.52	54.28	1.32	42.69	1.01
Kubanskij	CN30861	1	A	3	A	13	A	1	A	1	A	1	A	5.35	0.11	3.23	0.12	18.90	2.28	13.10	0.26	59.28	2.21	45.49	1.16
FP2270	FP2270	1	A	3	A	1	A	1	A	3	A	1	A	5.06	0.10	5.47	0.55	17.69	2.35	12.27	0.33	59.60	2.47	42.71	1.08
UGG5-5	UGG5-5	1	A	3	A	20	A	1	A	3	A	1	A	4.00	0.10	2.48	0.14	12.96	1.80	11.17	0.32	69.31	1.71	44.54	0.91
M5791	M5791	1	A	3	A	2	A	1	A	3	A	2	A	4.40	0.06	2.44	0.12	11.41	1.44	9.89	0.28	71.85	1.43	43.73	0.86

[1]Expressed as a percentage of the total fatty acid composition

[2]Standard error
