# Supplementary material for: Fatty acid composition and desaturase gene expression in flax (Linum usitatissimum L.)
Source: J Appl Genet. 2014 May 29;55(4):423–32. doi: 10.1007/s13353-014-0222-0 (PMC4185102; doi:10.1007/s13353-014-0222-0)
Supplement: Supplementary file 2 — Reverse transcriptase PCR of six fatty acid desaturase genes and the reference apt1 gene from six flax genotypes comparing the level of expression at 20 days after anthesis showing the consistent amplification of the control apt1 gene and the relative differential expression of the six desaturase genes. (PDF 22 kb) [file 13353_2014_222_MOESM2_ESM.pdf]

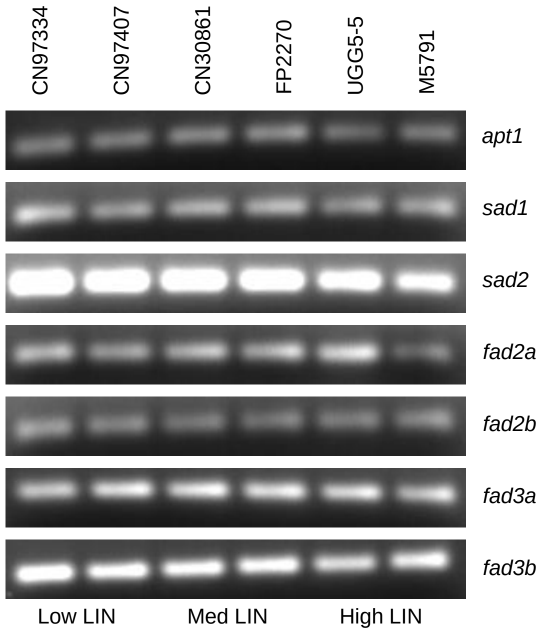

ESM_2. Reverse transcriptase PCR of six fatty acid desaturase genes and the reference *apt1* gene from six flax genotypes comparing the level of expression at 20 days after anthesis showing the consistent amplification of the control *apt1* gene and the relative differential expression of the six desaturase genes.
