# Supplementary material for: Fatty acid composition and desaturase gene expression in flax (Linum usitatissimum L.)
Source: J Appl Genet. 2014 May 29;55(4):423–32. doi: 10.1007/s13353-014-0222-0 (PMC4185102; doi:10.1007/s13353-014-0222-0)
Supplement: Supplementary file 3 — Analysis of variance for expression (gene:apt1) of six desaturase genes. Mean square values and statistical significance for sad1, sad2, fad2a, fad2b, fad3a and fad3b during seed development of six flax genotypes are shown. (PDF 92 kb) [file 13353_2014_222_MOESM3_ESM.pdf]

ESM_3. Analysis of variance for expression (*gene:apt1*) of six desaturase genes. Mean square values and statistical significance for *sad1, sad2*, *fad2a, fad2b*, *fad3a* and *fad3b* during seed development of six flax genotypes are shown**.**

**Gene(s)**	**Source of variation**	**Mean Square**	**Pr > F**
sad1, sad2, fad2a,	Gene	74.23	<.0001*
fad2b, *fad3a*, *fad3b*	Genotype	3.12	0.1400
	Stage	49.29	<.0001*
	Gene*Genotype	0.53	0.9998
	Gene*Stage	3.24	0.0101*
	Genotype*Stage	1.20	0.9306
	Gene*Genotype*Stage	0.27	1.0000
sad1	Genotype	0.35	0.9572
	Stage	4.27	0.0233
	Genotype*Stage	0.20	1.0000
sad2	Genotype	2.41	0.7873
	Stage	28.28	<.0001*
	Genotype*Stage	1.01	1.0000
fad2a	Genotype	0.31	0.8895
	Stage	8.88	<.0001*
	Genotype*Stage	0.27	0.9999
fad2b	Genotype	0.31	0.9147
	Stage	4.25	0.0013*
	Genotype*Stage	0.19	1.0000
fad3a	Genotype	1.50	0.2420
	Stage	11.83	<.0001*
	Genotype*Stage	0.36	0.9994
fad3b	Genotype	0.90	0.6805
	Stage	7.96	<.0001*
	Genotype*Stage	0.49	0.9992

* Statistical significance ($P < 0.01$)
