# Supplementary material for: Fatty acid composition and desaturase gene expression in flax (Linum usitatissimum L.)
Source: J Appl Genet. 2014 May 29;55(4):423–32. doi: 10.1007/s13353-014-0222-0 (PMC4185102; doi:10.1007/s13353-014-0222-0)
Supplement: Supplementary file 4 — P-value of sad1, sad2, fad2a, fad2b, fad3a and fad3b for genotypes during seed development. (PDF 89 kb) [file 13353_2014_222_MOESM4_ESM.pdf]

ESM_4. *P*-value of *sad1*, *sad2, fad2a, fad2b, fad3a* and *fad3b* for genotypes during seed development

**Seed developmental stage (DAA)**	***P*-value**					
	sad1	***sad2***	***fad2a***	***fad2b***	***fad3a***	***fad3b***
8	n.s.	n.s.	n.s.	n.s.	n.s.	n.s.
12	n.s.	n.s.	n.s.	n.s.	n.s.	n.s.
16	n.s.	n.s.	n.s.	n.s.	n.s.	n.s.
20	n.s.	n.s.	n.s.	n.s.	n.s.	n.s.
24	n.s.	n.s.	n.s.	n.s.	n.s.	n.s.
28	n.s.	n.s.	n.s.	n.s.	n.s.	n.s.
32	n.s.	n.s.	0.01*	n.s.	0.001*	0.002*

* Significant at $P < 0.01$; *n.s.* = non-significant
