# Supplementary material for: Fatty acid composition and desaturase gene expression in flax (Linum usitatissimum L.)
Source: J Appl Genet. 2014 May 29;55(4):423–32. doi: 10.1007/s13353-014-0222-0 (PMC4185102; doi:10.1007/s13353-014-0222-0)
Supplement: Supplementary file 5 — Nucleotide sequences of the promoter region of sad1, sad2, fad2a, fad2b, fad3a and fad3b genes. Sequence numbering is relative to the ATG codon (+1). 5′-UTR is highlighted and putative transcription initiation site (TSS) is indicated. The TATA and CAAT boxes are boxed. Putative cis-acting regulatory elements are underlined and designated with the names of each motif (Higo et al. 1999; Chang et al. 2008). Red colour letters indicate the single point mutations identified in the promoter regions of the corresponding genotype. (PDF 198 kb) [file 13353_2014_222_MOESM5_ESM.pdf]

sad1

-849 TTGCAGATCTCGTTGCTGATAACACATACATGGCTACAAGACTCTAAAACGGTTCAAAGTGAAATTGTTTTGGTGGTAGAGTTGTGTGTTTGGTGACTCG

ACGTBOX
-749 AAAGTTCTGGATTCGAATCCAGCATTCCCCACAAAATAGACACCAACGTAGTGTTTATTTACCGTCTTCTATCTTGTATTGACCGAGAGTTTCGATATAC

T(CN30861)
-649 TCCGACAAAAAAAGACATCTTCACATCATCAAATGGATCCGTAGTTAGTGCAGTGGCTCGAATAACATAAATGAAAAAAGGAAAAAATTTGCCTGAAATC

-549 GATGCTCAAAACAAGTAGAAATTCATTCAAACATATTTAGACAAACACGATCATTTAGCATCATCAAATTAATAACAAGAGCAAACAATAAAGCACATAG

-449 CAAAACATACAATAGTCGTCTTGCAATGTCATATGATAATAAGCCAGTGAAACCATGAAGCCCAAGTGAAGTGGTCAAGTGGAGCTGAAAGCTTCCGAAC

DPBFCORE MYBCORE
-349 CCAAGCCCCCGCTACCGGGTTAGGACATACGACACGCGACATGCTACGAAACTTAAAAATCGGTCACGCAGTTAATGGAACAAATGAAACGCAACGACTA

POLLEN1LELAT52 WRKY
-249 TTAAGTGACCATTTTGCAGAAATGATATGAAAAAGTGACCATTTAGACAAATGAGCAAAGAAAATACAAGTGGCGAGTGCTGACATAATAAACCGAATGC

DOFCORE HSE EBOX
-149 AGGCGTTACCATCCAATTTTACAACCATTCAATTCAAAAGTTTTTCCAATTTCCATTTCCTCATCTGCCTTACCCATAAATCTCGACGGACACCAAAAAA

TSS
-49 CTCAGCCAGCTTGCCCCCAAACAACAGCGCAGAAAAACCTTCAACAACAATG
+1

sad2

-954 AACATCAATGTCAATCTCTGCAGATTTTTGTTAGCAGCAGGTCATGATTCTTTTTTGGTTGATTCTTGTGAATGTAAGCTATTTGTTGTTGTAATATATG

-854 CATTGATTGTGATTTTGTTTTAGCTTTGATCAATGAAATAAATCTCGTTCAACCCAACCATCAGGCTCTTTCATATTCATTTTGACGACTATATATACAT

MYBCORE POLLEN1LELAT52
-754 AATCGTACAAACTATTCGGTTAACTAATCTACAGAAAGTCGGAGTTAGCTAGAGATTGTCAAGGAGGAGGAGATCATACACCTAATTTTGAAGCTGATTC

WRKY
-654 TTCATCTATGATTTCGAGTTTTGACTTGATTTGGCTCTTCGATATTCGAAATTAAATGCCTCAATGCCTCCAAAGTGCTCTCTACTTGCGGGTGGACCTA

EBOX ACGTBOX
-554 CAAAACTAGACAAACAGGTGCAAAAAACATGTGTTTACACGTCCATGTTATCTTGCATTGGCCCATGTTTTCTGCATTGTAAATCTTTCCCCAAACACAT

-454 AGTTAGACGAAGTCGATAATCTAGCACCATCAAATCAATAACACGAGCAAATAATAAAGTAAATAGTGAAACCATGAAGCCTAATTGGTCGAGTGGAGCT

DOFCORE DPBFCORE TSS
-354 GAAAGCTTTCATCGGTATCGAACCCAACCCCCCCTGCTACGAAACTTAAAAATGGGTTACGCAATTACACTCGATAGAACTGATGAAACGCAACGATTGT

-254 TAAGTAACCATTTTGCAGAAACGATAATTAACAAGTGACCATTTGGATAAATGACCAGAGAAAATACAAGTGGCGAGTGCTGACATAATAAACCGAATGC

-154 GGGCGTTACCATCCAATTTTACAACCATTCAATTCAATATCTCACATTCAAGTTTTTCCAACTTCCATTTCCTCATCTGCCTTACCCATAAATCTCGACA

A(CN30861)
-54 CCAAAACACTCAGCCAGCTTCGTCCCAAACAACGCAGAAAAACCTTCAACAACAATG
+1

fad2a

-702 ACACCCCTCCTGCATGCGCGAATCTGTGGGATTTTTCCTGCAATTGAAATTGATTTTCCGCTAATTAGGGGGTGTTTGGCTGAGTTCTTCGTTCACCAGT

POLLEN1LELAT52
-602 AGAAATTGCAAAACTGGAAATGAGAACACCAAAAATTGAATGAAAAAACTACATTAGCTGGTTTATTAAACGCCTGCCCTCCTTCATATTCTTCTTTTGG

EBOX MYBCORE
-502 GTTCCGGGTCCTAATCATATGCTGATTCAGTCAATTCTCTGTTGCTTTATTGGTGAAATTGGAAGGAATTCAAACTTTTGTTTGCTTCAGTGCGTGAATG

DOFCORE
-402 AATGAACCCACTCTATCACCTTCAGGGAAATCTTGGTGGGTTGGTAGTTAGGTGGTCATAGTTGGGTTTCTTTTCAAAGGTTGAAAAGAGGGTTCTTTTC

WRKY TSS
-302 ACAAGTTATTTGACTGGTGGAATCATTAACTAATTTGTTCCTAAAAATGGACAAGTTGTGCTAAATCACGCAACTGAAAACTGGAAAGGAAAGACCAATT

-202 CAAAATCGGGTAATTAGGTTTGGTGGTGATAATGTTAAGTAGGACTTTTGATGTAGGGGAAGAATCCAAGTAATCATTCCTTGTCAAGAATCCTTCTCTG

-102 TTTCTTCCTGCAAGTTATTGTGATAATTCTGATGAATCTTTTCTTCTGATCATATATGATATATATGCAGGTGCTGTAACAAAATACACAGAAAGAAGAA

-2 AA**ATG**
+1

fad2b

EBOX
-928 TCTTGATGCAATCTCTCTGAGTTTGAAAAAATATCTTTTTCTTTTGGAATGATTGATCGTCTCTTACTATTGTTTGAACATTACACGTGACATTGGTGTG

HSE
-828 ATGAACTGTAACAGGGCCAATAAGAGATTTGTTTCTGTGCTCTGAATTTTCTTTCTTTCTTTGAATCTGGAAAGTCAATAATACTGATATGTTACCCTAA

POLLEN1LELAT52 DOFCORE
-728 AGTGATCAATGGATTTTCTATTCTTAGAAAGCCTGATTTTTTTGGGGGAAAGGGTTCAGAATTGAGATTGCAACACTGTACCAAACATTGTATGTTATTC

-628 AATAGGGGTTAGATTCTGAGCTTCTTGGTCTGTGACTTTCATAAGAGAGAATTTAATTCTCGTAAATGGTCATTTTCAATTATACTTGCTGTGGCCTGCA

MYBCORE
-528 TATATCTACCTTGCCGCTATGCTTACAGCATGCTGTTATTTTGAATGTAACCTGATTTGTGGAATTTAGCTTACTTTTAAACTTACTAATAATTAGATTC

WRKY DPBFCORE TSS
-428 GTATCAGGATAGCACTACTTGCTTAAACTGACTATAAAAACACAAGTCTCCCATGGTTGTAC**G**CTTGTCCTGGTTCAATACGCAACTTCAGAGAGACATT

-328 TTTTCTGACGACGATTGATAAAGACGGTAAACTGTCTCGTGGAACCGAGAGTAGTTGTTTCCTCTCAAATTTGAACCTTAACGTTTCTGCATCGGATATT

-228 CTCGATTTGGGGAGGGCATTATCCTTGTTGTTACAGTATATCTATTGGTATCCTAGGCATTGTAAAATAAGATTGTCCTTTGTAACCTAAATTGTAACGG

-128 TTGTTTCCCAATACTCCAAACACCTCAACTCTGTGATTCTGCGCGCACACAGTTTCTTTCCATCACATCACAAACATGATTTTTTTCTCTGGTGCCAAAC

-28 TTCTGCAGGTGCTGTTGATAAAGCAAGA**ATG**
+1

fad3a

-853 AAGAGAGTTGTAAGAGTTTCGACTAAGTTCAAATGGAGCCCAAAGTTTGATCATCAGTTTGTGAAAACAAAGTCAAGCTCGTCCATATCTCTGCCTTGTT

HSE DPBFCORE
-753 CCCAACCCACTACATAGCATCTGGAAGACCTCGTACTTCACATTCTCGGACCGAAGGACAACCAATACCCCCCTTGTGATCCTAAACACATGCACAAATC

-653 CCTCTGCCCGAAACTTGCCCGAACTTACTCCCTAAGACCGATGCCCACTTGAGTCACATGAGTTGATTAGTCGATTTCACCCTAGCTCCCGCGAACTCAG

-553 CAGTGCCCGTTGCGACTCCGCCAAATCACTAATCCTTAATTAAAAGAACTAATAAGTTGATATCATCACATTTGTGGTAACTCATGCATGCACATAGGTT

EBOX
-453 TCCTAGATACCATTGAAGGAAGTTGCCATGTGTTTGAATCAAAGATTTGCCCACCACCATTGATACTGAAATTGAAGAACCTAGCAGCCAGCAACGGCTC

MYBCORE
-353 CTTTTCATTTGTCTTTCAACAGAGCAAGTAACAACAACCGTTGCCTAAACTGAAACCCAATAAAGAGCAAAAAAAAAGGGGTTGGGTGGTGTAGGCTAGT

-253 TTGTCTGAAATCAGTGTACATTTTGCATTTCCATTTACTCTTCTCCATCCACTTGGCATCCTGCATTACTTCTTCTTCGTTAGTTCTCACCAACCTACAT

DOFCORE
-153 ACTCTTCGGTTATAAATACTGTGAGGCTGAAACCAAAGGCCACTCAGTCTATTCATTATTATTCAAAAATATATATTGGGTTTGTTTGGTGCAGATTACA

WRKY TSS
-53 GTGACTTCAAAACTGTGGCTCTGCA**C**GACCAAACTATGAGCCCTCCAAACTCA**ATG**
+1

fad3b

ACGTBOX (ACGT), WRKY (TGAC), HSE (CCAAT)
-780 CCCAACCCATTACATGACGTCAGAAAGAGCTCGTACTTCACATTCTCGGACCGAAGGACAACCATGCTTTGTTGACATCTCCGAAAGGCAACCAATTATA

EBOX (CACATG)
-680 CCCCCTAGTGATCCTAAACACATGCACATGTCCCTATGCCCGAACTTGCTCCCTAAATATTGCAGCATCAACATTGCAATTCAGAGTCCCCCAAACAAGA

-580 CCGATGCCCACTTGAGCCACATTAGTTGATTAGTCGATTTCGCCCTAGCTCCCGCTAACCGATCGTAGCCACTCCACCAAGTCACTAATCCTTGATTAAA

DPBFCORE (ACACCTG)
-480 GAGTTAAACAAGTTGATATCACACCTGTGGTAACTCATGCACATACCATTGAAGAAAGTTGCCATGTGTTAGAATCAAGGATTTGCCCACCACCATTGAT

MYBCORE (CCGTT)
-380 ACTGAAATTGAAGAATGCTAGCTAGCAGGCAGCAACGGCTCCTTTTCATTTGTCTTTCAACAGAGCAAGTAACAACAACCGTTGCCTAAACTGAAACCCA

POLLEN1LELAT52 (AGAAA)
-280 ATAAAGAGCAGAAAAAAGGGTTGGGTGGTGTAGGCTAGTTTGTCTGAAATCAATGTACATTTTGCACTTCCATTTACTCTTCTCCATCCACTTGGCATCC

A(CN30861) (at **T**), DOFCORE (AAAG), TSS (**A**)
-180 TACATTATTACTTCTTCTTCGTTAGCTCTCACCAAACTTTACATACAC**T**TTTTCGGTTATAAATACTGTGAGCCTCAAAGCAAAGGCCACTC**A**CTCTATT

-80 CATTATTATTAAAAAAAAAATATTGGTTGTTTGGTGCAGATTATAGTGACTTCAAAACTGTGGCTCTGCAGGACCAAACT**ATG**
+1

ESM_5. Nucleotide sequences of the promoter region of *sad1*, *sad2*, *fad2a*, *fad2b*, *fad3a* and *fad3b* genes. Sequence numbering is relative to the ATG codon (+1). 5'-UTR is highlighted and putative transcription initiation site (TSS) is indicated. The TATA and CAAT boxes are boxed. Putative *cis*-acting regulatory elements are underlined and designated with the names of each motif (Higo et al. 1999; Chang et al. 2008). Red colour letters indicate the single point mutations identified in the promoter regions of the corresponding genotype.
