# Supplementary material for: Fatty acid composition and desaturase gene expression in flax (Linum usitatissimum L.)
Source: J Appl Genet. 2014 May 29;55(4):423–32. doi: 10.1007/s13353-014-0222-0 (PMC4185102; doi:10.1007/s13353-014-0222-0)
Supplement: Supplementary file 6 — Analysis of variance for fatty acid composition for the data collected from six environments. Mean square values and statistical significance for palmitic acid (PAL), stearic acid (STE), oleic acid (OLE), linoleic acid (LIO) and linolenic acid (LIN) are shown. (PDF 10 kb) [file 13353_2014_222_MOESM6_ESM.pdf]

ESM_6. Analysis of variance for fatty acid composition for the data collected from six environments. Mean square values and statistical significance for palmitic acid (PAL), stearic acid (STE), oleic acid (OLE), linoleic acid (LIO) and linolenic acid (LIN) are shown.

**Source of variation**	**PAL**	**STE**	**OLE**	**LIO**	**LIN**
Genotype (G)	3.96*	6.48*	63.43*	280.06*	299.00*
Location (L)	6.00*	59.75*	4399.06*	89.49*	4492.78*
Year (Y)	6.88*	15.56*	715.02*	46.41*	444.19*
G * L	0.21*	0.24*	2.95*	5.46*	6.32*
G * Y	0.25*	0.18*	2.74*	4.99*	7.96*
L * Y	0.24*	15.75*	878.42*	25.58*	822.69*
G * L * Y	0.15*	0.11*	2.08*	3.60*	5.43*

* Statistical significance ($P < 0.0001$)
